# Application of the simple atrial fibrillation better care pathway for integrated care management in frail patients with atrial fibrillation: A nationwide cohort study

**DOI:** 10.1002/joa3.12364

**Published:** 2020-06-05

**Authors:** Pil‐Sung Yang, Jung‐Hoon Sung, Eunsun Jang, Hee Tae Yu, Tae‐Hoon Kim, Gregory Y. H. Lip, Boyoung Joung

**Affiliations:** ^1^ Department of Cardiology CHA Bundang Medical Center CHA University Seongnam Republic of Korea; ^2^ Division of Cardiology Department of Internal Medicine Severance Cardiovascular Hospital Yonsei University College of Medicine Seoul Republic of Korea; ^3^ Liverpool Centre for Cardiovascular Science University of Liverpool and Liverpool Heart and Chest Hospital Liverpool United Kingdom

**Keywords:** atrial fibrillation, frailty, integrated management, mortality

## Abstract

**Background:**

The benefit of integrated care management was unknown in frail atrial fibrillation (AF) patients. This study evaluated whether compliance with the atrial fibrillation Better Care (ABC) pathway for integrated care management would improve clinical outcomes in frail AF patients.

**Methods:**

From the Korea National Health Insurance Service database, 262,987 nonvalvular AF patients were enrolled between 1 January 2005 and 31 December 2015. For each patient, the Hospital Frailty Risk Score and category were calculated retrospectively using all available ICD‐10 diagnostic codes. Patients were divided into three frailty‐based risk categories: low (<5 points, n = 221,542), intermediate (5‐15 points, n = 37,341), and high risk (>15 points, n = 4,104).

**Results:**

Over a mean follow‐up of 5.9 (interquartile range 3.2, 9.4) years, in high frailty risk patients, the ABC group had lower rates of all‐cause death (6.5 vs 17.5 per 100 person‐years, *P* < .001; hazard ratio [HR] 0.74; 95% confidence interval [CI] 0.56‐0.97) but was nonsignificant for the composite outcome (10.5 vs 26.0 per 100 person‐years, *P* = .101; HR 0.79; 95% CI 0.59‐1.05) compared with the Non‐ABC group. When the three frailty categories were compared, the greatest benefit on mortality was seen in the high frailty group (p_int_ < 0.001), but for the composite outcome, there was no statistical interaction for the three frailty categories (p_int_ = 0.063).

**Conclusions:**

Compliance with the simple ABC pathway is associated with improved outcomes in AF patients with high frailty risk. Given the high healthcare burden associated with frail AF patients, integrated AF management should be implemented to improve outcomes in these patients.

## Graphical abstract

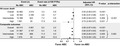

## 1 INTRODUCTION

Atrial fibrillation (AF) is the most common sustained cardiac arrhythmia among elderly individuals,^1^, ^2^, ^3^ and it has enormous socioeconomic implications given the risk of mortality and morbidity resulting from stroke, congestive heart failure, dementia, and impaired quality of life.^1^, ^2^, ^3^, ^4^, ^5^ Frailty is also associated with more adverse clinical outcomes in elderly individuals admitted to the hospital. AF may be a marker of frailty in elderly individuals and may be related to a loss of independence in performing activities of daily living.^6^ AF could worsen the state of frailty, and patients with AF could have 4‐fold increased odds of being classified as frail, compared with patients without AF.^7^

Recent trials involving AF^8^, ^9^ revealed a high (4.6% per year) rate of all‐cause death in patients with AF, with only one out of 10 deaths related to stroke and approximately five or six out of 10 deaths related to cardiovascular causes. Therefore, a more integrated and holistic approach beyond anticoagulation therapy for patients with AF has been advocated in guidelines to reduce mortality and adverse outcomes in AF.^10^, ^11^, ^12^ One way is to streamline management approaches that would be applicable across the entire AF patient pathway, starting with primary care and linking with secondary care (even for cardiologists and noncardiologists), and be understandable for patients with AF. The ABC (atrial fibrillation better care) pathway has been proposed as a simple, integrated approach.^13^ This pathway streamlines the care pathway as follows: “A” Avoid stroke with Anticoagulation; “B” Better symptom management (ie, patient‐centered, symptom‐directed decisions on rate vs rhythm control); and “C” Cardiovascular and comorbidity management, including lifestyle factors.^13^

Application of the simple ABC pathway was associated with a lower risk of adverse outcomes in patients with AF in a post hoc analysis of a clinical trial cohort as well as other AF cohorts.^14^, ^15^, ^16^ However, the population‐based benefit on clinical outcomes owing to a compliance with the ABC pathway has not been previously evaluated in patients with AF at a high frailty risk. Give the close association between AF and frailty, this study aimed to evaluate whether compliance with the ABC pathway would improve population‐based clinical outcomes in patients with AF belonging to different frailty risk categories, using a nationwide AF cohort.

## 2 METHODS

### 2.1 Data source

We performed a retrospective analysis of data from the national health claims database (NHIS‐2016‐4‐026) established by the National Health Insurance Service (NHIS) of Korea.^1^, ^2^, ^3^ The NHIS is a single insurer managed by the Korean government, and the majority (97.1%) of the Korean population are mandatory subscribers, with the remaining 3% of the population being medical aid subjects. Since 2006, information regarding Medical Aid beneficiaries has been incorporated into a single NHIS database. Therefore, the data extracted from the NHIS claims database are based on the entire Korean population. The NHIS claims database includes diagnoses, procedures, biochemical test results, prescription records, and demographic information. The database is open to researchers whose study protocols are approved by official review committees. This study was approved by the Institutional Review Board of Yonsei University Health System (4‐2016‐0179).

### 2.2 Study cohort

**FIGURE 1:**
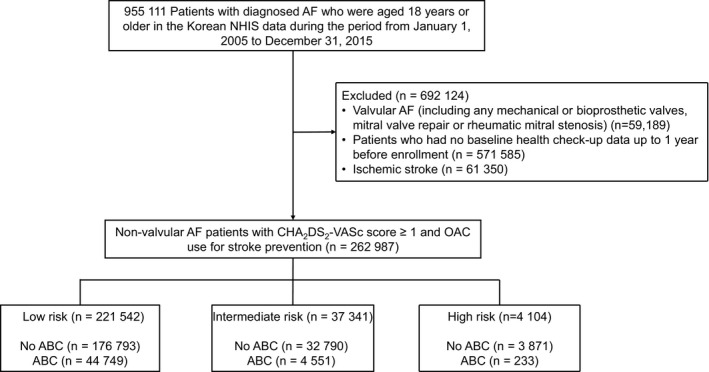
Flowchart of study population enrolment and analyses. AF, atrial fibrillation; OAC, oral anticoagulant; NHIS, National Health Insurance Service

From the Korean NHIS database, a total of 955 111 patients with prevalent AF who were aged 18 years or older were identified from 1 January 2005 to 31 December 2015. Patients with valvular AF, such as those with any mechanical or bioprosthetic heart valves, mitral valve repair, or rheumatic mitral stenosis (n = 59 189); those without baseline health check‐up data up to 1 year before enrolment (n = 571 585); and those who had ischemic stroke (n = 61 350) were excluded. Finally, a total of 262,987 patients with nonvalvular AF were enrolled in the study to evaluate the impact of the ABC pathway on the long‐term clinical outcomes of these patients (Figure 1).

For each patient, the Hospital Frailty Risk Score was calculated retrospectively using all available ICD‐10 diagnostic codes that were documented for the particular admission, as recommended by Gilbert et al.^17^ The score is an aggregate of 109 ICD‐10 diagnostic codes found to be associated with frailty‐based risk (Supplementary Table S1). Each diagnostic code was assigned a specific value proportional to how strongly it predicted frailty. According to the aggregate score, patients were divided into three frailty‐based risk categories: low risk (<5 points, n = 221 542), intermediate risk (5‐15 points, n = 37 341), and high risk (>15 points, n = 4104).^17^

### 2.3 Definition of the ABC pathway‐compliant group

The integrated care group (ABC group) was defined according to the criteria summarized in Supplementary Figure S1. “A” was defined as the use of oral anticoagulants, in accordance with guidelines, with high adherence (prescription covering ≥80% of days); “B” was defined in relation to visits requiring medical contact with outpatient clinics (<5 visits per year during the follow‐up period); “C” was defined as optimal management of the main cardiovascular comorbidities (hypertension, heart failure, myocardial infarction, peripheral artery disease, stroke/transient ischemic attack [TIA], diabetes mellitus, and obesity). Optimal management of hypertension was defined as baseline blood pressure values <140/90 mmHg. For obesity, body mass index less than 30 kg/m^2^ was considered optimal management. For other comorbidities, appropriate use of cardiovascular prevention medications according to current guidelines was considered optimal management. Patients who fulfilled all criteria were defined as the “ABC” group, and those who did not fulfill all criteria were defined as the “non‐ABC” group.

### 2.4 Comorbidities and endpoints

AF was identified using International Classification of Disease, Tenth Revision (ICD‐10) codes: I48. To ensure accuracy, the diagnosis was established based on more than one or two outpatient records of ICD‐10 codes in the database. The diagnosis of AF has previously been validated in the NHIS database, with a positive predictive value of 94.1%.^1^, ^4^, ^18^, ^19^ Comorbidities were identified from ICD‐10 codes and prescription, as in previous studies (Supplementary Table S2).^18^

The primary clinical outcomes of this study were all‐cause death, ischemic stroke, heart failure admission, acute myocardial infarction, major bleeding, and a composite outcome of these five outcomes. Any diagnosis of ischemic stroke in the emergency room or inpatient clinic with concomitant brain imaging studies, including computed tomography or magnetic resonance imaging, was defined as incident ischemic stroke. The accuracy of the diagnosis of ischemic stroke in the NHIS claims data has been previously validated.^4^ The other definitions of clinical outcomes are presented in Supplementary Table S2. Patients were followed from the index date until the study outcomes occurred or up to the end of follow‐up, whichever occurred first.

### 2.5 Statistical analysis

Categorical data are reported as proportions, while continuous data are reported as medians with interquartile ranges (IQRs). The categorical variables were compared using Fisher's exact test or Pearson chi‐square test, and continuous variables were compared using Student's *t* test. The main analyses compared the clinical outcomes between the ABC (ie, integrated care) and non‐ABC groups. Incidence rates were defined as events per 100 person‐years at risk but expressed as annualized percentage rates for comprehensiveness. The relationships between the total number of ABC criteria fulfilled and the clinical outcomes were also investigated.

The cumulative incidences of adverse outcomes were presented using Kaplan–Meier curves and compared across the groups using the log‐rank test. Using Cox proportional hazard regression model, the hazard ratios (HRs) for adverse outcomes according to the use of integrated care (ABC) were analyzed. Clinical variables including age, gender, heart failure, hypertension, diabetes mellitus, previous myocardial infarction, peripheral artery disease, economic status, CHA_2_DS_2_‐VASc, and HAS‐BLED score were adjusted for HR. *P*‐values < 05 were considered significant. Statistical analyses were conducted using SAS version 9.3 (SAS Institute) and R version 3.3.2 (The R Foundation, www.R‐project.org).

## 3 RESULTS

### 3.1 Baseline characteristics

Comparisons between the ABC and non‐ABC groups are presented in Supplementary Table S3. Compared with the non‐ABC group, patients in the ABC group were less likely to be female (39.4% vs 38.6%, *P* = .001) and had a lower mean age (65.0 [IQR 56.0, 72.0] vs 50.0 [IQR 41.0, 58.0], *P* < .001). Compared with patients in the non‐ABC group, those in the ABC group had a lower prevalence of comorbidities, such as hypertension, heart failure, diabetes mellitus, stroke/TIA, hemorrhage stroke, vascular disease, chronic kidney disease, and dyslipidemia. In addition, patients in the ABC group had a lower mean CHA_2_DS_2_‐VASc score than that of patients in the non‐ABC group.

The baseline characteristics between patients with AF with different hospital frailty risk categories who were compliant and noncompliant with the ABC pathway are compared in Table 1. Multivariable analysis showed that in patients with high frailty risk, factors including age ≥65 years and female gender were independently associated with a likelihood of being compliant with the ABC pathway. In contrast, high CHA_2_DS_2_‐VASc score, hypertension, liver disease, or history of intracranial bleeding were related to noncompliance with the ABC pathway. In patients with intermediate and low frailty risk, heart failure, and vascular disease were also related to compliance with the ABC pathway (Table 2).

**TABLE 1:** Comparison of baseline characteristics between patients with atrial fibrillation and different hospital frailty risks who were compliant with and not compliant with the ABC pathway

Characteristics	Low frailty risk			Intermediate frailty risk			High frailty risk		
	No‐ABC (N = 176 793)	ABC (N = 44,749)	*P*‐value	No‐ABC (N = 32 790)	ABC (N = 4,551)	*P*‐value	No‐ABC (N = 3871)	ABC (N = 233)	*P*‐value
Female	38.1%	38.3%	.522	45.2%	41.3%	<.001	48.5%	42.5%	.088
Age, y	65 (50, 71)	50 (41, 57)	<.001	69 (60, 75)	53 (44, 60)	<.001	73 (63, 79)	58 (49, 64)	<.001
Age ≥ 65	50.2%	5.9%	<.001	65.1%	12.5%	<.001	72.8%	23.6%	<.001
Age ≥ 75	14.5%	1.6%	<.001	28.0%	4.4%	<.001	41.6%	10.3%	<.001
Economic status	12 (5, 17)	13 (6, 17)	<.001	13 (5, 17)	12 (6,16)	<.001	12 (5,17)	9 (3,15)	<.001
CHA_2_DS_2_‐VASc score	2 (1, 3)	0 (0, 1)	<.001	3 (2, 4)	1 (0, 1)	<.001	4 (2, 5)	1 (0, 2)	<.001
mHAS‐BLED score†	2 (1, 3)	0 (0, 1)	<.001	2 (1, 3)	0 (0, 1)	<.001	3 (2, 3)	1 (0, 1)	<.001
Hospital Frailty Risk Score	0 (0, 1.8)	0 (0, 1.6)	<.001	7.4 (6, 9.7)	6.9 (5.7, 8.9)	<.001	18 (16, 22)	18 (16, 20)	<.001
Charlson comorbidity index	2 (1, 4)	1 (0, 2)	<.001	4 (2, 6)	2 (1, 3)	<.001	6 (4, 9)	3 (2, 6)	<.001
Hypertension	64.2%	6.5%	<.001	70.9%	11.2%	<.001	78.4%	20.2%	<.001
Heart failure	22.6%	1.6%	<.001	29.4%	2.5%	<.001	38.3%	5.6%	<.001
Diabetes mellitus	17.9%	2.1%	<.001	29.8%	4.7%	<.001	42.8%	10.3%	<.001
Previous ischemic stroke/ TIA	0.0%	0.0%	—	0.0%	0.0%	—	0.0%	0.0%	—
Previous MI	6.2%	0.4%	<.001	11.4%	0.9%	<.001	16.2%	1.7%	<.001
PAOD	8.5%	0.7%	<.001	12.8%	1.6%	<.001	17.1%	1.3%	<.001
Vascular disease	13.8%	1.0%	<.001	21.9%	2.3%	<.001	29.3%	2.6%	<.001
Hypertrophic cardiomyopathy	1.2%	0.6%	<.001	1.0%	0.5%	<.001	0.7%	0.9%	<.001
Chronic kidney disease	2.3%	0.7%	<.001	7.7%	1.8%	<.001	15.6%	5.2%	<.001
Liver disease	36.1%	28.0%	<.001	48.9%	38.8%	<.001	60.2%	50.6%	.005
Malignant neoplasm	17.4%	14.0%	<.001	31.8%	22.5%	<.001	41.5%	33.0%	.014
Hyperthyroidism	8.2%	7.3%	<.001	10.6%	8.4%	<.001	12.8%	7.3%	.019
Hypothyroidism	6.8%	5.5%	<.001	10.1%	6.9%	<.001	13.3%	12.0%	.643
Venous thromboembolism	2.4%	1.8%	<.001	5.9%	4.0%	<.001	11.4%	9.4%	.413
COPD	11.1%	3.6%	<.001	22.1%	7.1%	<.001	31.5%	15.5%	<.001
Previous intracranial bleeding	0.4%	0.2%	<.001	2.6%	1.8%	.002	9.3%	2.6%	.001
History of any bleeding	3.9%	2.5%	<.001	20.0%	13.7%	<.001	40.3%	27.5%	<.001
Coagulation/ platelet defect	2.2%	1.5%	<.001	8.1%	5.4%	<.001	16.4%	14.2%	.424
Osteoporosis	22.8%	10.1%	<.001	41.5%	19.1%	<.001	55.7%	34.8%	<.001
Medications									
OAC, baseline	4.0%	3.3%	<.001	4.7%	6.1%	<.001	6.1%	18.1%	<.001
OAC, follow‐up	30.9%	22.1%	<.001	26.8%	35.5%	<.001	21.1%	58.5%	<.001
Antiplatelet, baseline	53.1%	14.2%	<.001	58.5%	22.9%	<.001	65.1%	38.3%	<.001
Antiplatelet, follow‐up	68.3%	31.6%	<.001	58.1%	25.7%	<.001	48.3%	31.8%	<.001
Statin	25.2%	7.2%	<.001	34.0%	16.4%	<.001	41.6%	33.1%	<.001
Beta blocker	34.2%	7.2%	<.001	39.9%	13.0%	<.001	47.3%	25.7%	<.001
ACE inhibitor/ARB	38.4%	5.1%	<.001	47.9%	12.6%	<.001	58.3%	28.6%	<.001
Diuretics	38.2%	5.2%	<.001	49.0%	13.3%	<.001	59.1%	30.7%	<.001
Digoxin	8.5%	1.3%	<.001	6.9%	1.2%	<.001	7.3%	2.1%	<.001

Note

Values are presented as median (Q1, Q3, quartiles [25th and 75th percentiles]) or %. †Modified HAS‐BLED = hypertension, 1 point: >65 years old, 1 point: stroke history, 1 point: bleeding history or predisposition, 1 point: liable international normalized ratio, not assessed: ethanol or drug abuse, 1 point: drug predisposing to bleeding, 1 point.

Abbreviations: ACE, angiotensin converting enzyme; ARB, angiotensin II receptor blocker; COPD, chronic obstructive pulmonary disease; MI, myocardial infarction; OAC, oral anticoagulant; PAOD, peripheral artery occlusive disease; TIA, transient ischemic attack.

**TABLE 2:** Factors associated with compliance to the ABC pathway in different frailty categories

	Low frailty risk		Intermediate frailty risk		High frailty risk	
	HR (95% CI)	*P*‐value	HR (95% CI)	*P*‐value	HR (95% CI)	*P*‐value
Age ≥ 65 y	1.07 (1.05‐1.09)	<.001	—	—	1.06 (1.04‐1.08)	<.001
Age ≥ 75 y	1.21 (1.19‐1.23)	<.001	1.12 (1.11‐1.13)	<.001	—	—
Female	1.17 (1.15 ‐1.19)	<.001	1.09 (1.08‐1.10)	<.001	1.05 (1.04‐1.07)	<.001
Economic status	1.00 (1.00‐1.00)	<.001	1.00 (1.00‐1.00)	<.001	—	—
CHA_2_DS_2_‐VASc score	0.93 (0.91‐0.95)	<.001	0.97 (0.96‐0.97)	<.001	0.98 (0.97‐0.99)	<.001
HAS‐BLED score	—	—	1.02 (1.01‐1.02)	<.001	—	—
Hospital Frailty Risk Score	1.00 (1.00‐1.00)	<.001	—	—	—	—
Charlson comorbidity Index	1.00 (1.00‐1.00)	.005	—	—	—	—
Heart failure	1.05 (1.03‐1.07)	<.001	1.02 (1.01‐1.03)	<.001	—	—
Hypertension	0.85 (0.84‐0.87)	<.001	0.88 (0.87‐0.89)	<.001	0.94 (0.92‐0.96)	<.001
Diabetes	1.03 (1.02‐1.05)	<.001	—	—	—	—
Previous MI	1.03 (1.02‐1.05)	<.001	—	—	—	—
Vascular disease	1.05 (1.03‐1.07)	<.001	1.02 (1.01‐1.04)	<.001	‐	‐
Liver disease	0.99 (0.98‐0.99)	<.001	0.97 (0.96‐0.97)	<.001	0.97 (0.96‐0.99)	.002
Malignant neoplasm	1.02 (1.01‐1.02)	<.001	—	—	—	—
Hyperthyroidism	0.99 (0.98‐0.99)	<.001	0.99 (0.98‐1.00)	.017	—	—
Venous thromboembolism	1.01 (1.00‐1.02)	.031	—	—	—	—
Intracranial bleeding	0.97 (0.95‐0.99)	.006	0.97 (0.95‐0.99)	<.001	0.96 (0.94‐0.99)	.003
History of bleeding	—	—	0.99 (0.98‐1.00)	.004	0.98 (0.97‐1.00)	.013
COPD	1.01 (1.00‐1.01)	.008	—	—	—	—
Osteoporosis	0.99 (0.99‐1.00)	<.001	—	—	—	—
OAC use	1.10 (1.09‐1.11)	<.001	1.08 (1.06‐1.10)	<.001	1.05 (1.02‐1.09)	.005
NOAC use	—	—	1.37 (1.01‐1.87)	.044	—	—
Antiplatelet use	1.04 (1.03‐1.05)	<.001	1.03 (1.01‐1.04)	<.001	—	—
Statin use	1.01 (1.00‐1.01)	.005	—	—	—	—
ACE inhibitor/ARB use	1.02 (1.01‐1.02)	<.001	1.01 (1.00‐1.02)	.005	—	—
Diuretics use	1.02 (1.02‐1.03)	<.001	1.02 (1.01‐1.02)	<.001	—	—
Digoxin use	1.01 (1.01‐1.02)	<.001	—	—	—	—

Abbreviations: CI, confidence interval; HR, hazard ratio; NOAC nonvitamin K antagonist oral anticoagulant. Other abbreviations are same as in Table 1.

### 3.2 Death and composite outcomes

**FIGURE 2:**
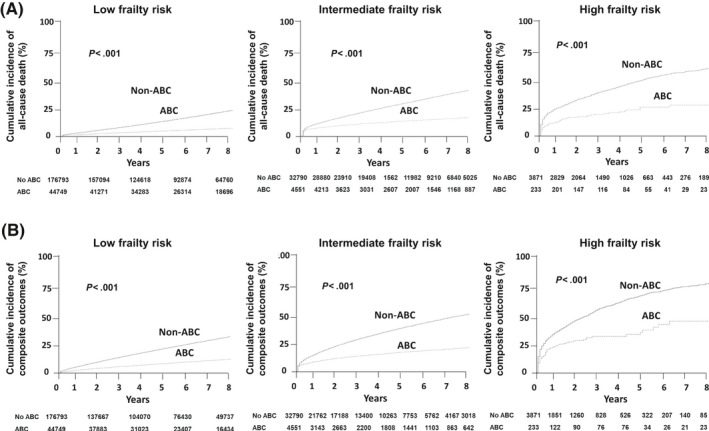
Cumulative incidences of all‐cause death (A) and composite outcomes (B) according to the use of integrated care (ABC) in patients with low (left panels), intermediate (mid panels), and high frailty risk (right panels)

**FIGURE 3:**
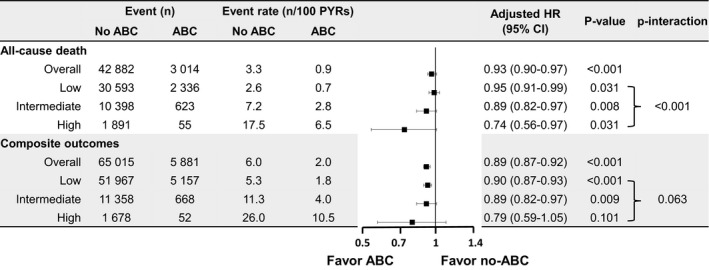
Events, event rates, risk of all‐cause death, and composite outcomes according to the use of integrated care (ABC) in patients with different frailty risks. CI, confidence interval; HR, hazard ratio; PYRs, person‐years

Patients with all three types of frailty risk in the ABC group had significantly lower cumulative incidences of all‐cause death (Figure 2A). During the mean follow‐up period of 5.9 (IQR 3.2, 9.4) years, compared with the non‐ABC group, the ABC group had lower rates of all‐cause death in the overall cohort (0.9 vs 3.3 per 100 person‐years, *P* < .001), and in the low (0.7 vs 2.6 per 100 person‐years, *P* < .001), intermediate (2.8 vs 7.2 per 100 person‐years, *P* < .001), and high frailty risk groups (6.5 vs 17.5 per 100 person‐years, *P* < .001) (Figure 3). After adjusting for clinical variables, compared with the non‐ABC group, the ABC group had a significantly lower risk of all‐cause death in the overall cohort (adjusted HR 0.93; 95% CI 0.90‐0.97, *P* < .001), and in the low (adjusted HR 0.95; 95% CI 0.91‐0.99, *P* = .031), intermediate (adjusted HR 0.89; 95% CI 0.82‐0.97, *P* = .008), and high frailty risk groups (adjusted HR 0.74; 95% CI 0.56‐0.97, *P* = .031) (Figure 3). There was a significant statistical interaction for the three frailty categories, with the greatest benefit observed in the high frailty group (*P*_int_ < 0.001) (Figure 3).

The ABC group had significantly lower cumulative incidences of composite outcomes in all three frailty risk groups (Figure 2B). During follow‐up, the ABC group had lower rates of composite outcomes in the overall cohort (2.0 vs 6.0 per 100 person‐years, *P* < .001; adjusted HR 0.89; 95% CI 0.87‐0.92, *P* < .001), and in the low (1.8 vs 5.3 per 100 person‐years, *P* < .001; adjusted HR 0.90; 95% CI 0.87‐0.93, *P* < .001) and intermediate (4.0 vs 11.3 per 100 person‐years, *P* = .009; adjusted HR 0.89; 95% CI 0.82‐0.97, *P* = .009) frailty risk groups (Figure 3). With regard to the composite outcomes in the high frailty group, there was no significant difference between the ABC and non‐ABC groups (10.5 vs 26.0 per 100 person‐years, *P* = .101; HR 0.79; 95% CI 0.59‐1.05, *P* = .101), and there was no statistically significant interaction for the three frailty categories (p_int_ = 0.063) (Figure 3).

### 3.3 Other outcomes

**FIGURE 4:**
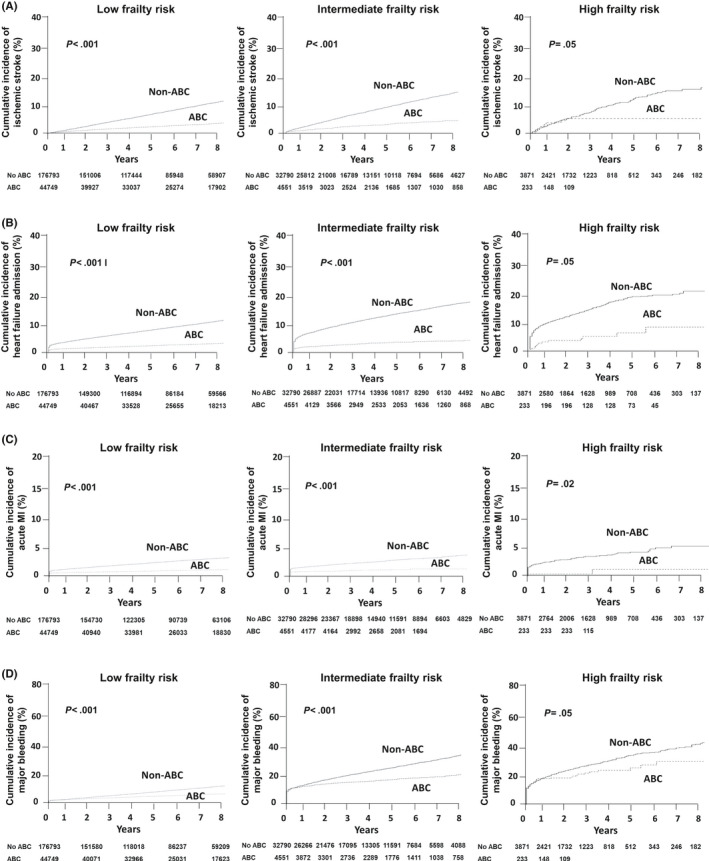
Cumulative incidences of ischemic stroke (A), heart failure admission (B), acute myocardial infarction (C), and major bleeding (D) according to use of integrated care (ABC) in patients with low (left panels), intermediate (mid panels), and high frailty risk (right panels). MI, myocardial infarction

The Kaplan–Meier cumulative incidence curves for other outcomes are presented in Figure 4. Among AF patients with low and intermediate frailty risk, lower cumulative incidences of ischemic stroke, heart failure admission, acute myocardial infarction, and major bleeding were observed in the ABC group compared with the non‐ABC group (all log‐rank *P* < .001). Among AF patients with high frailty risk, the lower cumulative incidence of clinical outcomes was also observed in the ABC group than in the non‐ABC group, but statistical significance was on the border.

**FIGURE 5:**
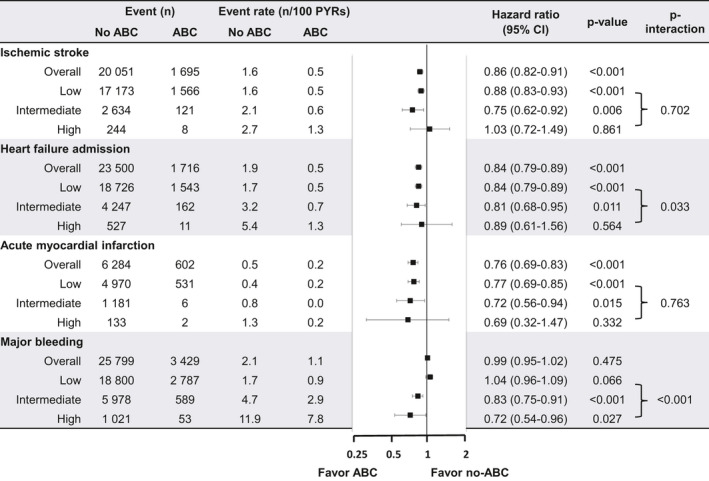
Events, event rates, and risks of other adverse outcomes according to the use of integrated care (ABC) in patients with different frailty risks. CI, confidence interval; HR, hazard ratio; PYRs, person‐years

The event rates and risks for other outcomes according to the ABC and non‐ABC groups are presented in Figure 5. Compared with the non‐ABC group, the ABC group had lower event rates and risk of ischemic stroke, heart failure admission, and acute myocardial infarction in overall, low and intermediate frailty risk groups (all *P* < .05). But among AF patients with high frailty risk group, there was no significant difference in risks of ischemic stroke, heart failure admission, and acute myocardial infarction between ABC and non‐ABC groups. Compared with the non‐ABC group, the ABC group had lower event rates and risk of major bleeding among patients with intermediate and high frailty risk, with a statistically significant interaction (*P* < .001). But among AF patients with overall and low frailty risk group, there was no significant difference in the risk of major bleeding between ABC and non‐ABC groups.

## 4 DISCUSSION

In this largest nationwide analysis of patients with AF according to frailty risk, the event rates and risks of the all‐cause death, ischemic stroke, heart failure admission, acute myocardial infarction, major bleeding, and composite of these outcomes were significantly lower in the ABC group than in the non‐ABC group. In addition, among patients with high frailty risk, compared with patients in the non‐ABC group, those in the ABC group had lower rates of all‐cause death; however, the composite outcome was nonsignificantly lower in the ABC group. When the three frailty categories were compared, the greatest benefit on mortality was observed in the high frailty group; however, with regard to the composite outcome, there was no statistical interaction for the three frailty categories. Given the close association between AF and frailty and the high healthcare burden associated with AF, a streamlined holistic approach to the management of AF would improve outcomes in such patients.

### 4.1 Mortality and outcomes according to frailty risk

The use of an integrated care approach to AF management has been associated with reduced cardiovascular hospitalization and all‐cause mortality.^20^ Nevertheless, approaches for providing integrated care have varying complexity.^11^ There is a need to have a simple, practical, and easily operational method to streamline the decision‐making management approaches to allow uniform applicability across the entire AF patient pathway, linking primary care and secondary care (including cardiologist and noncardiologists), and to be understandable for patients with AF, facilitating their engagement.

The ABC pathway was proposed to streamline the interventions and decision‐making, and to optimize the patient management pathway, providing simple guidance for the main components of integrated care.^13^ Recent AF management guidelines have incorporated the ABC pathway.^12^, ^21^ Nevertheless, there are limited data on the value of the ABC pathway in high‐risk patient groups. In this study, we show that the ABC pathway was related to reduced mortality and composite outcomes in frail patients with AF. The strong impact of the ABC pathway on overall mortality substantiates and strengthens the concept that a holistic approach for integrated management is associated with a significant clinical benefit for patients with AF. Indeed, compliance to the ABC pathway was also associated with a lower risk of ischemic stroke, heart failure admission, and acute myocardial infarction, as well as major bleeding, in patients with AF. A greater benefit in terms of major bleeding was observed in patients with AF and high frailty risk.

Although the risk of thromboembolic events is high, the rate of adequate oral anticoagulation is lower in frail patients with AF compared to nonfrail patients.^8^, ^9^, ^10^ However, specific suggestions on oral anticoagulant (OAC) use have been based on advanced age and/or the presence of various comorbid disease states (eg, age > 75, renal impairment, prior history of bleeding), but not on frailty.^11^, ^12^ It may be appropriate to use no anticoagulation to avoid bleeding events in severe frail patients with AF. A recent US cohort study has shown that rivaroxaban but not apixaban or dabigatran was associated with reduced stroke versus warfarin in frail patients with nonvalvular AF while no significant difference in bleeding versus warfarin.^22^ Therefore, “A” (Avoid stroke with Anticoagulation) as an integrated approach in patients with AF at a high frailty risk may not be always associated with good outcomes.

### 4.2 Study limitations

This retrospective population‐based observational study was performed using nationwide cohort data and should be interpreted in the context of the following limitations. First, the baseline diagnoses of AF, comorbidities, and clinical outcomes were dependent on the diagnostic codes registered by the physicians; therefore, these could be inaccurate, although the method for diagnosis has been validated in previous studies, and our internal validation found a high correlation with the actual diagnosis of AF.^4^ Second, patients included in the non‐ABC group appeared more complex from a clinical perspective, with multiple comorbidities; however, compared with the non‐ABC group, the ABC group had adjusted lower risk of all‐cause death and composite outcome in all subgroups regardless of age, gender, and comorbidities. Conversely, given the high prevalence of comorbidities in the non‐ABC group, we can speculate that full implementation of the ABC pathway may reduce the risk even further. Third, in this study based on claims data, we used adherence (≥80%) as a surrogate of OAC optimization use, but fully recognize the limitations of lack of time in therapeutic range (TTR) data in patients with vitamin K antagonist use, or the availability of label‐adherence prescribing. Fourth, this study defined the low frequency of medical contact as “B” (better symptom management). However, a symptom is just one of the factors to decide the frequency of medical contact. Our definition using the frequency of medical contact alone may not have sufficiently reflected “B” (better symptom management) of AF patients. Fifth, this study included some patients who have undergone catheter ablation for eliminating AF. In Castle AF trial, ablation for AF in patients with heart failure improved all‐caused death. Hence, ablation may influence the improvement of outcomes of patients. Finally, in this study, we enrolled only East Asian patients, and therefore, whether the results can be extrapolated to other ethnic populations remains uncertain. Despite these limitations, to the best of our knowledge, this study presents the largest nationwide population dataset available in the literature to investigate the relationship between frailty and cardiovascular outcomes in patients with AF.

## 5 CONCLUSIONS

Compliance with the simple ABC pathway is associated with improved outcomes in patients with AF who have a high frailty risk. Given the high healthcare burden associated with AF, such a streamlined holistic approach to the management of AF should be implemented to improve outcomes in such patients.

## CONFLICT OF INTEREST

GYHL: Consultant for Bayer/Janssen, BMS/Pfizer, Medtronic, Boehringer Ingelheim, Novartis, Verseon, and Daiichi‐Sankyo, and speaker for Bayer, BMS/Pfizer, Medtronic, Boehringer Ingelheim, and Daiichi‐Sankyo. No fees were directly received personally. BJ: Speaker for Bayer, BMS/Pfizer, Medtronic, and Daiichi‐Sankyo. Research fund from Medtronic and Abbott. None of the other authors have anything to disclose.

## AUTHOR CONTRIBUTIONS

PSY, JHS, and BJ designed the study, conducted the data analysis, and wrote the manuscript; GYHL designed the study and revised the manuscript. EJ participated in data analysis; HTY and THK participated in data collection. All authors have reviewed and approved the final version of the manuscript.

## Supporting information

Supplementary Material
