# Supplementary material for: Application of the simple atrial fibrillation better care pathway for integrated care management in frail patients with atrial fibrillation: A nationwide cohort study
Source: J Arrhythm. 2020 Jun 5;36(4):668–77. doi: 10.1002/joa3.12364 (PMC7411200; doi:10.1002/joa3.12364)
Supplement: Supplementary file 1 — Supplementary Material [file JOA3-36-668-s001.docx]

**Supporting Information**

**Supplementary Table 1.** Calculation of the hospital Frailty risk score in patients with different Hospital Frailty Risk Categories. The score is an aggregate of 109 ICD-10 diagnostic codes that were found to be associated with frailty risk.^1^ According to the aggregate scores, patients were divided into the three frailty risk categories low risk (<5 points), intermediate risk (5–15 points) and high risk (>15 points).

| ICD Code | Points awarded | ICD Description | Hospital Frailty Risk Categories | | |
| --- | --- | --- | --- | --- | --- |
|  |  |  | Low (n=67,827) | Intermediate (n=42,679) | High (n=18,537) |
| K59 | 1.8 | Other functional intestinal disorders | 8,761 (12.9) | 21,100 (49.4) | 14,447 (77.9) |
| G81 | 4.4 | Hemiplegia | 11 (0) | 6,200 (14.5) | 7,865 (42.4) |
| N39 | 3.2 | Other disorders of urinary system (includes urinary tract infection and urinary incontinence) | 1,019 (1.5) | 6,936 (16.3) | 8,544 (46.1) |
| F00 | 7.1 | Dementia in Alzheimer's disease | 0 (0) | 1,222 (2.9) | 5,854 (31.6) |
| I69 | 3.7 | Sequelae of cerebrovascular disease (secondary codes) | 530 (0.8) | 5,208 (12.2) | 7,635 (41.2) |
| E87 | 2.3 | Other disorders of fluid, electrolyte and acid base balance | 2,141 (3.2) | 9,794 (22.9) | 9,129 (49.2) |
| I63 | 0.8 | Cerebral Infarction | 10,257 (15.1) | 18,362 (43) | 12,823 (69.2) |
| M81 | 1.4 | Osteoporosis without pathological fracture | 3,281 (4.8) | 9,805 (23) | 8,595 (46.4) |
| J18 | 1.1 | Pneumonia, organism unspecified | 4,763 (7) | 9,721 (22.8) | 8,038 (43.4) |
| I67 | 2.6 | Other cerebrovascular diseases | 978 (1.4) | 4,158 (9.7) | 3,847 (20.8) |
| S06 | 2.4 | Intracranial injury | 1,147 (1.7) | 4,372 (10.2) | 3,613 (19.5) |
| M25 | 2.3 | Other joint disorders, not elsewhere classified | 1,081 (1.6) | 4,111 (9.6) | 4,248 (22.9) |
| R31 | 3.0 | Unspecified hematuria | 675 (1) | 3,084 (7.2) | 2,975 (16) |
| A09 | 1.1 | Diarrhea and gastroenteritis of presumed infectious origin | 3,720 (5.5) | 7,607 (17.8) | 6,006 (32.4) |
| S22 | 1.8 | Fracture of rib(s), sternum and thoracic spine | 1,116 (1.6) | 4,316 (10.1) | 3,884 (21) |
| G40 | 1.5 | Epilepsy | 658 (1) | 3,466 (8.1) | 5,044 (27.2) |
| M79 | 1.1 | Other soft tissue disorders, not elsewhere classified | 2,030 (3) | 5,265 (12.3) | 5,128 (27.7) |
| R94 | 1.4 | Abnormal results of function studies | 2,510 (3.7) | 4,228 (9.9) | 2,769 (14.9) |
| M19 | 1.5 | Other arthrosis | 1,154 (1.7) | 3,704 (8.7) | 3,692 (19.9) |
| S00 | 3.2 | Superficial injury of head | 305 (0.4) | 1,909 (4.5) | 1,713 (9.2) |
| F03 | 2.1 | Unspecified dementia | 207 (0.3) | 1,679 (3.9) | 3,532 (19.1) |
| L03 | 2.0 | Cellulitis | 911 (1.3) | 2,456 (5.8) | 2,258 (12.2) |
| S32 | 1.4 | Fracture of lumbar spine and pelvis | 886 (1.3) | 3,508 (8.2) | 3,530 (19) |
| R55 | 1.8 | Syncope and collapse | 1,240 (1.8) | 2,936 (6.9) | 1,925 (10.4) |
| N17 | 1.8 | Acute renal failure | 629 (0.9) | 2,505 (5.9) | 2,808 (15.1) |
| G45 | 1.2 | Transient cerebral ischemic attacks and related syndromes | 1,856 (2.7) | 3,669 (8.6) | 2,922 (15.8) |
| F05 | 3.2 | Delirium, not induced by alcohol and other psychoactive substances | 82 (0.1) | 912 (2.1) | 1,958 (10.6) |
| S01 | 1.1 | Open wound of head | 2,117 (3.1) | 3,615 (8.5) | 2,379 (12.8) |
| L89 | 1.7 | Decubitus ulcer | 220 (0.3) | 1,528 (3.6) | 3,442 (18.6) |
| F01 | 2.0 | Vascular dementia | 150 (0.2) | 1,061 (2.5) | 3,176 (17.1) |
| R11 | 0.3 | Nausea and vomiting | 7,195 (10.6) | 12,552 (29.4) | 8,532 (46) |
| E86 | 2.3 | Volume depletion | 398 (0.6) | 1,682 (3.9) | 1,526 (8.2) |
| E05 | 0.9 | Thyrotoxicosis [hyperthyroidism] | 2,858 (4.2) | 3,776 (8.8) | 2,389 (12.9) |
| M48 | 0.5 | Spinal stenosis (secondary code only) | 3,621 (5.3) | 7,227 (16.9) | 5,245 (28.3) |
| R56 | 2.6 | Convulsions, not elsewhere classified | 148 (0.2) | 1,096 (2.6) | 1,733 (9.3) |
| F32 | 0.5 | Depressive episode | 1,930 (2.8) | 5,817 (13.6) | 7,292 (39.3) |
| N18 | 1.4 | Chronic renal failure | 677 (1) | 2,139 (5) | 2,059 (11.1) |
| N28 | 1.3 | Other disorders of kidney and ureter, not elsewhere classified | 1,095 (1.6) | 2,291 (5.4) | 1,811 (9.8) |
| S80 | 2.0 | Superficial injury of lower leg | 416 (0.6) | 1,491 (3.5) | 1,382 (7.5) |
| R26 | 2.6 | Abnormalities of gait and mobility | 91 (0.1) | 822 (1.9) | 1,609 (8.7) |
| R00 | 0.7 | Abnormalities of heart beat | 2,866 (4.2) | 3,819 (8.9) | 2,498 (13.5) |
| A41 | 1.6 | Other septicemia | 321 (0.5) | 1,574 (3.7) | 2,014 (10.9) |
| K26 | 1.6 | Duodenal ulcer | 831 (1.2) | 1,761 (4.1) | 1,294 (7) |
| K92 | 0.8 | Other diseases of digestive system | 1,487 (2.2) | 2,915 (6.8) | 2,836 (15.3) |
| R47 | 1.0 | Speech disturbances, not elsewhere classified | 546 (0.8) | 2,256 (5.3) | 2,702 (14.6) |
| S72 | 1.4 | Fracture of femur | 345 (0.5) | 1,480 (3.5) | 2,029 (10.9) |
| D64 | 0.4 | Other anemia | 2,014 (3) | 5,183 (12.1) | 4,910 (26.5) |
| S42 | 2.3 | Fracture of shoulder and upper arm | 279 (0.4) | 912 (2.1) | 892 (4.8) |
| R40 | 2.5 | Somnolence, stupor and coma | 70 (0.1) | 650 (1.5) | 1,194 (6.4) |
| R41 | 2.7 | Other symptoms and signs involving cognitive functions and awareness | 66 (0.1) | 606 (1.4) | 1,071 (5.8) |
| I95 | 1.6 | Hypotension | 350 (0.5) | 1,171 (2.7) | 1,249 (6.7) |
| R13 | 0.8 | Dysphagia | 215 (0.3) | 1,607 (3.8) | 3,550 (19.2) |
| G30 | 4.0 | Alzheimer's disease | 8 (0) | 188 (0.4) | 865 (4.7) |
| E16 | 1.4 | Other disorders of pancreatic internal secretion | 397 (0.6) | 1,242 (2.9) | 1,275 (6.9) |
| A04 | 1.1 | Other bacterial intestinal infections | 381 (0.6) | 1,313 (3.1) | 1,902 (10.3) |
| M80 | 0.8 | Osteoporosis with pathological fracture | 522 (0.8) | 1,946 (4.6) | 2,080 (11.2) |
| G20 | 1.8 | Parkinson's disease | 115 (0.2) | 561 (1.3) | 1,227 (6.6) |
| N20 | 0.7 | Calculus of kidney and ureter | 1,407 (2.1) | 1,807 (4.2) | 993 (5.4) |
| G31 | 1.2 | Other degenerative diseases of nervous system, not elsewhere classified | 165 (0.2) | 871 (2) | 1,280 (6.9) |
| R63 | 0.9 | Symptoms and signs concerning food and fluid intake | 375 (0.6) | 1,267 (3) | 1,341 (7.2) |
| J69 | 1.0 | Pneumonitis due to solids and liquids | 176 (0.3) | 845 (2) | 1,603 (8.6) |
| B96 | 2.9 | Other bacterial agents as the cause of diseases classified to other chapters (secondary code) | 54 (0.1) | 323 (0.8) | 517 (2.8) |
| J96 | 1.5 | Respiratory failure, not elsewhere classified | 167 (0.2) | 669 (1.6) | 851 (4.6) |
| N19 | 1.6 | Unspecified renal failure | 113 (0.2) | 444 (1) | 655 (3.5) |
| K52 | 0.3 | Other noninfective gastroenteritis and colitis | 1,455 (2.1) | 2,545 (6) | 2,386 (12.9) |
| E53 | 1.9 | Deficiency of other B group vitamins | 91 (0.1) | 371 (0.9) | 526 (2.8) |
| R50 | 0.1 | Fever of unknown origin | 3,656 (5.4) | 6,844 (16) | 5,631 (30.4) |
| F10 | 0.7 | Mental and behavioral disorders due to use of alcohol | 464 (0.7) | 957 (2.2) | 766 (4.1) |
| H91 | 0.9 | Other hearing loss | 424 (0.6) | 676 (1.6) | 563 (3) |
| E83 | 0.4 | Disorders of mineral metabolism | 524 (0.8) | 1,298 (3) | 1,388 (7.5) |
| M15 | 0.4 | Polyarthrosis | 418 (0.6) | 1,155 (2.7) | 1,370 (7.4) |
| R29 | 3.6 | Other symptoms and signs involving the nervous and musculoskeletal systems (R29.6 Tendency to fall) | 7 (0) | 109 (0.3) | 178 (1) |
| R33 | 1.3 | Retention of urine | 125 (0.2) | 328 (0.8) | 354 (1.9) |
| Z93 | 1.0 | Artificial opening status | 85 (0.1) | 257 (0.6) | 550 (3) |
| E55 | 1.0 | Vitamin D deficiency | 123 (0.2) | 326 (0.8) | 398 (2.1) |
| Z22 | 1.7 | Carrier of infectious disease | 107 (0.2) | 200 (0.5) | 116 (0.6) |
| R32 | 1.2 | Unspecified urinary incontinence | 50 (0.1) | 166 (0.4) | 333 (1.8) |
| L08 | 0.4 | Other local infections of skin and subcutaneous tissue | 286 (0.4) | 650 (1.5) | 660 (3.6) |
| R79 | 0.6 | Other abnormal findings of blood chemistry | 161 (0.2) | 482 (1.1) | 421 (2.3) |
| R54 | 2.2 | Senility | 8 (0) | 80 (0.2) | 198 (1.1) |
| Z50 | 2.1 | Care involving use of rehabilitation procedures | 10 (0) | 54 (0.1) | 223 (1.2) |
| S51 | 0.5 | Open wound of forearm | 331 (0.5) | 440 (1) | 376 (2) |
| B95 | 1.7 | Streptococcus and staphylococcus as the cause of diseases classified to other chapters | 22 (0) | 105 (0.2) | 192 (1) |
| R45 | 1.2 | Symptoms and signs involving emotional state | 57 (0.1) | 160 (0.4) | 209 (1.1) |
| M41 | 0.9 | Scoliosis | 79 (0.1) | 213 (0.5) | 246 (1.3) |
| S09 | 1.2 | Other and unspecified injuries of head | 72 (0.1) | 191 (0.4) | 134 (0.7) |
| H54 | 1.9 | Blindness and low vision | 29 (0) | 77 (0.2) | 102 (0.6) |
| R02 | 1.0 | Gangrene, not elsewhere classified | 56 (0.1) | 149 (0.3) | 178 (1) |
| L97 | 1.6 | Ulcer of lower limb, not elsewhere classified | 12 (0) | 102 (0.2) | 121 (0.7) |
| W01 | 0.9 | Fall on same level from slipping, tripping and stumbling | 40 (0.1) | 159 (0.4) | 150 (0.8) |
| J22 | 0.7 | Unspecified acute lower respiratory infection | 87 (0.1) | 180 (0.4) | 168 (0.9) |
| W18 | 2.1 | Other fall on same level | 7 (0) | 60 (0.1) | 56 (0.3) |
| R44 | 1.6 | Other symptoms and signs involving general sensations and perceptions | 21 (0) | 50 (0.1) | 67 (0.4) |
| W19 | 3.2 | Unspecified fall | 2 (0) | 22 (0.1) | 20 (0.1) |
| R69 | 1.3 | Unknown and unspecified causes of morbidity | 12 (0) | 45 (0.1) | 34 (0.2) |
| T83 | 2.4 | Complications of genitourinary prosthetic devices, implants and grafts | 3 (0) | 13 (0) | 21 (0.1) |
| Z87 | 1.5 | Personal history of other diseases and conditions | 10 (0) | 23 (0.1) | 15 (0.1) |
| Z75 | 2.0 | Problems related to medical facilities and other health care | 2 (0) | 18 (0) | 16 (0.1) |
| X59 | 1.5 | Exposure to unspecified factor | 5 (0) | 22 (0.1) | 18 (0.1) |
| W10 | 0.9 | Fall on and from stairs and steps | 9 (0) | 38 (0.1) | 24 (0.1) |
| U80 | 0.8 | Agent resistant to penicillin and related antibiotics | 2 (0) | 28 (0.1) | 49 (0.3) |
| Z99 | 0.8 | Dependence on enabling machines and devices | 3 (0) | 17 (0) | 42 (0.2) |
| Y95 | 1.2 | Nosocomial condition | 2 (0) | 14 (0) | 21 (0.1) |
| W06 | 1.1 | Fall involving bed | 3 (0) | 6 (0) | 12 (0.1) |
| Y84 | 0.7 | Other medical procedures as the cause of abnormal reaction of the patient | 2 (0) | 9 (0) | 11 (0.1) |
| Z91 | 0.5 | Personal history of risk-factors, not elsewhere classified | 1 (0) | 5 (0) | 10 (0.1) |
| Z74 | 1.1 | Problems related to care-provider dependency | 1 (0) | 1 (0) | 3 (0) |
| Z73 | 0.6 | Problems related to life-management difficulty | 3 (0) | 4 (0) | 1 (0) |
| Z60 | 1.8 | Problems related to social environment | 0 (0) | 0 (0) | 0 (0) |

**Supplementary Table 2.** Definitions and ICD-10 codes used for defining the comorbidities and clinical outcomes.

|  | **Definitions** | **ICD-10 codes or conditions** |
| --- | --- | --- |
| **Comorbidities** |  |  |
| Hypertension ^2-4^ | Defined from diagnosis* plus treatment | I10, I11, I12, I13, I15  Treatment: all kinds of blood pressure lowering medications (>1 month). |
| Heart failure ^2-4^ | Defined from diagnosis* | I11.0, I50, I97.1 |
| Diabetes mellitus ^2-4^ | Defined from diagnosis* plus treatment | E10, E11, E12, E13, E14  Treatment: all kinds of oral antidiabetics and insulin. |
| Previous ischemic stroke ^2-4^ | Defined from diagnosis* | I63, I64 |
| Previous TIA ^2-4^ | Defined from diagnosis* | G45 |
| Previous myocardial infarction ^5^ | Defined from diagnosis* | I21, I22, I25.2 |
| Peripheral arterial occlusive disease ^2-4^ | Defined from diagnosis* | I70, I71 |
| Hypertrophic cardiomyopathy ^6^ | Defined from diagnosis | I42.1, I42.2 |
| Chronic kidney disease ^2-4^ | Defined from eGFR or diagnosis* (if laboratory value was not available, diagnosis code was used) | eGFR <60mL/min per 1.73 m^2^  N18, N19 |
| Dyslipidemia ^2-4^ | Defined from diagnosis* | E78 |
| Liver disease | Defined from diagnosis of chronic liver disease, cirrhosis, and hepatitis | B18, K70, K71, K72, K73, K74, K76.1 |
| Malignancy | Defined from diagnoses of cancer (non-benign) | C00-C97 |
| Hyperthyroidism | Defined from diagnosis* | E05 |
| Hypothyroidism | Defined from diagnosis* | E03 |
| Venous thromboembolism | Defined from diagnosis* | I80.1, I80.2, I80.3, I80.8, I80.9, I81, I82, I26 |
| Chronic obstructive pulmonary disease ^7^ | Defined from diagnosis* plus treatment | J42, J43(except J43.0), J44  Treatment: SABA, SAMA, LABA, LAMA, ICS, ICS+LABA, or methylxanthine (>1 months). |
| Previous intracranial bleeding ^2-4^ | Defined from diagnosis* | I60, I61, I62 |
| Coagulation or platelet defect | Defined from diagnosis* | D65, D66, D67, D68 (except D68.3), D69 (except D69.2) |
| Osteoporosis ^8^ | Defined from diagnosis* | M80, M81, M82 (except M82.0) |
| **Clinical outcomes** |  |  |
| Ischemic stroke ^2-4^ | Defined from admission diagnosis of ischemic stroke with concomitant imaging studies of the brain or related death | I63, I64  Brain-imaging studies include computed tomography or magnetic resonance imaging. |
| Heart failure admission | Defined from admission diagnosis (including only main and first sub-diagnosis) | I11.0, I50, I97.1 |
| Acute myocardial infarction ^2-4^ | Defined from admission diagnosis of acute myocardial infarction with concomitant use of dual antiplatelet therapy or related death | I21, I22 |
| Major bleeding ^2-4^ | Intracranial hemorrhage (ICH), gastrointestinal bleeding, or anemia caused by bleeding | I60-I62, K25-28 (subcodes 0-2 and 4-6 only), K92.0, K92.1, K92.2, K62.5, I85.0, I98.3, D62 |

*To ensure accuracy, comorbidities were established based on one inpatient or two outpatient records of ICD-10 codes in the database.

eGFR, estimated glomerular filtration rate; ESRD, end stage renal disease; ICD-10, International Classification of Diseases-10th Revision; TIA, transient ischemic attack. All covariates were validated in cited references

**Supplementary Table 3.** Comparison of baseline characteristics between atrial fibrillation patients compliant with and without ABC pathway.

| **Characteristics** | **Non-ABC**  **(N=213,454)** | **ABC**  **(N=49,533)** | **p-value** |
| --- | --- | --- | --- |
| Female | 39.4% | 38.6% | 0.001 |
| Age, years | 65.0 [56.0;72.0] | 50.0 [41.0;58.0] | <0.001 |
| Age ≥ 65 years | 52.9% | 6.6% | <0.001 |
| Age ≥ 75 years | 17.1% | 1.9% | <0.001 |
| Economic status | 12.0 [ 5.0;17.0] | 13.0 [ 6.0;17.0] | <0.001 |
| CHA_2_DS_2_-VASc score | 2.0 [ 1.0; 3.0] | 0.0 [ 0.0; 1.0] | <0.001 |
| mHAS-BLED score* | 2.0 [ 1.0; 3.0] | 0.0 [ 0.0; 1.0] | <0.001 |
| Hospital Frailty Risk Score | 0.7 [ 0.0; 3.4] | 0.0 [ 0.0; 2.2] | <0.001 |
| Charlson comorbidity index | 3.0 [ 1.0; 4.0] | 1.0 [ 0.0; 2.0] | <0.001 |
| Hypertension | 65.5% | 7.0% | <0.001 |
| Heart failure | 23.9% | 1.7% | <0.001 |
| Diabetes mellitus | 20.2% | 2.4% | <0.001 |
| Previous ischemic stroke / TIA | 0.0% | 0.0% | - |
| Previous MI | 7.1% | 0.4% | <0.001 |
| PAOD | 9.3% | 0.8% | <0.001 |
| Vascular disease | 15.3% | 1.1% | <0.001 |
| Hypertrophic cardiomyopathy | 1.2% | 0.6% | <0.001 |
| Chronic kidney disease | 3.4% | 0.8% | <0.001 |
| Liver disease | 38.5% | 29.1% | <0.001 |
| Malignant neoplasm | 20.0% | 14.9% | <0.001 |
| Hyperthyroidism | 8.7% | 7.4% | <0.001 |
| Hypothyroidism | 7.5% | 5.6% | <0.001 |
| Venous thromboembolism | 3.1% | 2.0% | <0.001 |
| COPD | 13.1% | 3.9% | <0.001 |
| Intracranial bleeding | 0.9% | 0.4% | <0.001 |
| History of bleeding | 7.0% | 3.7% | <0.001 |
| Coagulation or platelet defect | 3.4% | 1.9% | <0.001 |
| Osteoporosis | 26.2% | 11.0% | <0.001 |
| Medications |  |  |  |
| OAC | 3.3% | 2.6% | <0.001 |
| NOAC | 0.0% | 0.1% | 0.117 |
| Aspirin | 37.4% | 5.9% | <0.001 |
| P2Y12 inhibitor | 5.4% | 0.8% | <0.001 |
| Statin | 23.9% | 6.5% | <0.001 |
| Beta blocker | 32.9% | 6.8% | <0.001 |
| ACE inhibitor/ARB | 37.1% | 4.3% | <0.001 |
| Diuretics | 37.7% | 4.9% | <0.001 |
| Digoxin | 7.9% | 1.2% | <0.001 |

Values are presented as % or medians (interquartile ranges).

*Modified HASBLED = hypertension, 1 point; >65 years old, 1 point; stroke history, 1 point; bleeding history or predisposition, 1 point; liable international normalized ratio, not assessed; ethanol or drug abuse, 1 point; drug predisposing to bleeding, 1 point.

ACE, angiotensin converting enzyme; ARB, angiotensin II receptor blocker; COPD, Chronic obstructive pulmonary disease; MI, myocardial infarction; NOAC, non-vitamin K oral anticoagulants; OAC, oral anticoagulation; PAOD, peripheral artery occlusive disease; TIA, transient ischemic attack.

**Supplementary Figure 1.** The ABC pathway for integrated care management.

**
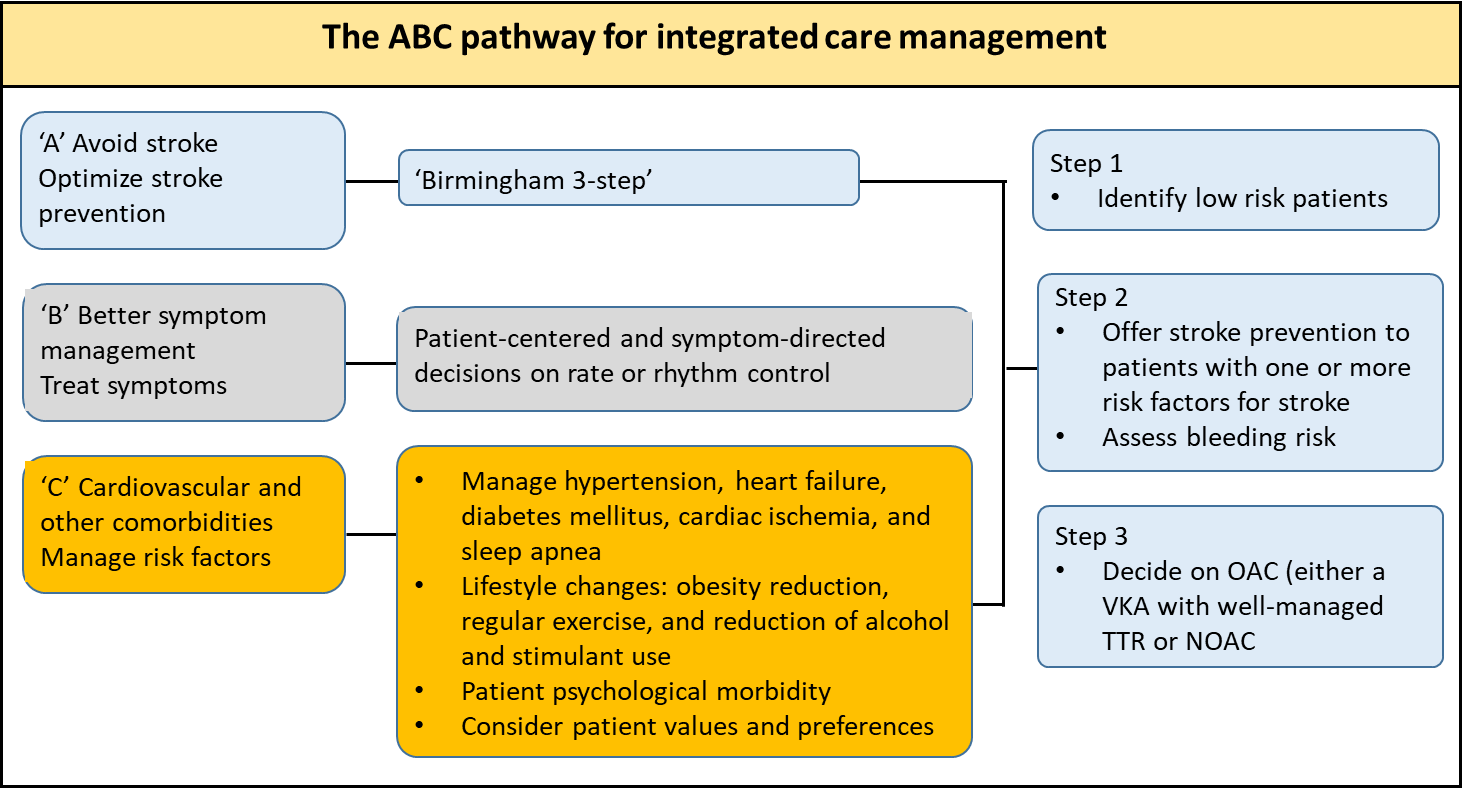
**

**References**

1. Gilbert T, Neuburger J, Kraindler J et al. Development and validation of a Hospital Frailty Risk Score focusing on older people in acute care settings using electronic hospital records: an observational study. Lancet 2018;391:1775-1782.

2. Lee H, Kim TH, Baek YS, et al. The Trends of Atrial Fibrillation-Related Hospital Visit and Cost, Treatment Pattern and Mortality in Korea: 10-Year Nationwide Sample Cohort Data. Korean Circ J 2017;47:56-64.

3. Kim TH, Yang PS, Kim D et al. CHA2DS2-VASc Score for Identifying Truly Low-Risk Atrial Fibrillation for Stroke: A Korean Nationwide Cohort Study. Stroke 2017;48:2984-2990.

4. Kim D, Yang PS, Jang E et al. Increasing trends in hospital care burden of atrial fibrillation in Korea, 2006 through 2015. Heart 2018.

5. Lee HY, Yang PS, Kim TH et al. Atrial fibrillation and the risk of myocardial infarction: a nation-wide propensity-matched study. Sci Rep 2017;7:12716.

6. Jung H, Yang PS, Sung JH,et al. Hypertrophic Cardiomyopathy in patients with Atrial Fibrillation: Prevalence and stroke risks in a nationwide cohort study. Thrombosis and Haemostasis. 2019;119:285-293.

7. Song S, Yang PS, Kim TH et al. Relation of Chronic Obstructive Pulmonary Disease to Cardiovascular Disease in the General Population. Am J Cardiol 2017;120:1399-1404.

8. Kim D, Yang PS, Kim TH, et al. Effect of Atrial Fibrillation on the Incidence and Outcome of Osteoporotic Fracture - A Nationwide Population-Based Study. Circulation journal. 2018;82:1999-2006.
